# The wound severity of animal bite victims visiting rabies prevention clinics and the influencing factors in Central China: a cross-sectional investigation

**DOI:** 10.1186/s12889-021-12207-4

**Published:** 2021-11-19

**Authors:** Dandan Li, Hanlin Liao, Fan Chen, Qingqing Jiang, Tiantian Wang, Zuxun Lu, Qiaoyan Liu, Shiyi Cao

**Affiliations:** 1 School of Public Health, Tongji Medical College, Huazhong University of Science and Technology, Wuhan, China; 2 Department of Medical Services Section, Taihe Hospital, Hubei University of Medicine, Shiyan, China; 3 Research Institute of Rehabilitation Information, China Rehabilitation Science Institute, Beijing, China; 4 China Rehabilitation Research Center, Beijing, China

**Keywords:** Exposure category, Animal bites, Associated factors, Rabies, China

## Abstract

**Background:**

Animal bites are a major public health problem. The more serious the bite wound is, the higher the risk of developing rabies is. This study aimed to investigate the severity of wounds among animal bite victims and identify the influencing factors in Wuhan, China.

**Methods:**

A cross-sectional study was conducted among 1015 animal bite victims visiting rabies prevention clinics. We performed a face-to-face interview to collect information on the exposure category of the bite wound, the type of the offending animal, exposure-to-risk situations, etc. Factors associated with exposure categories were identified by multivariate logistic regression analysis.

**Results:**

Five hundred and sixty-four (55.57%) cases were category III exposures, 418 (41.18%) were category II exposures, and 33 (3.25%) were category I exposures. People who were hurt by their own domestic animals (odds ratio [OR] = 1.55, 95% confidence interval [CI]: 1.14–2.10), and those exposed to animals unvaccinated against rabies (OR = 1.45, 95% CI: 1.08–1.95) had a higher risk for category III exposures. Respondents who did not know the fatality of rabies were more likely to be injured seriously compared to those who knew that rabies is fatal, and the OR was 1.40 (95% CI, 1.05–1.86).

**Conclusions:**

This study showed that factors associated with the severity of bite wounds mainly included types of the offending animal, vaccination status of the animal, and knowledge of rabies fatality. Educational programs and awareness-raising campaigns should be provided to decrease severe animal bites, especially targeting pet owners and those with limited rabies knowledge.

## Introduction

Animal bites and bite-associated diseases are considered to be serious health and economic problems all over the world. Animal bites may cause secondary infections, permanent disfigurement, disability, and rabies [1–4]. It is reported that 60–80% of animal bites are dog-related, 20–30% are cat-related, and bites by other animals such as rabbits, hamsters, and rats are much rarer [2]. Worldwide canine rabies causes approximately 59,000 human deaths, over 3.7 million disability-adjusted life years, and 8.6 billion USD economic losses annually [5]. Preventing animal bites is imperative.

Bite injuries range in severity from superficial abrasions, lacerations, and crush wounds to degloving injuries with major tissue loss, sometimes extending to the underlying bone [2]. The more serious the bite wound is, the higher the probability of occurring adverse events is. The World Health Organization (WHO) classifies the bite wound into three categories according to its severity and recommends wound treatment and rabies vaccination for category II and category III exposures as well as rabies immunoglobulin administration for category III exposures [6].

Previous studies reported the incidence of bite injuries [7–10] and the risk factors for animal bites [11, 12]. However, few studies concentrated on the severity of bite wounds. Although animal bites are preventable injuries, sometimes it may be difficult to avoid animal attacks for individuals who are pet owners and those who like playing with animals. Given that the serious bite wound could bring a negative effect on the victim’s daily life and mental health and generally has a higher risk of developing rabies if the person was bitten by a rabid animal, it is necessary to focus on the severity of bite wounds and identify the associated factors, which may provide an opportunity to reduce such health impairment.

China is an endemic country for rabies. Epidemiological data indicated that more than 40 million people were bitten or scratched in China every year [13]. The high prevalence of animal bites in China makes it essential to carry out animal bite researches. The present study aimed to collect information on the exposure category of animal bite victims and identify the influencing factors for the severity of bite wounds. The findings of the current investigation may help in developing and implementing practical strategies to reduce animal bites.

## Methods

### Sampling design and data collection

This is a cross-sectional survey. Data were collected from March 2016 to May 2016 in the city of Wuhan, which is one of the five largest pet cities in China, with more than 130,000 domestic animals. A multistage sampling technique was used to select participants. There were 15 districts in Wuhan, and three districts were selected by simple random sampling. Within each district, two rabies prevention clinics (RPCs) were randomly selected. Information on animal bite victims’ demographic characteristics, their animal injury history, and their knowledge of rabies was collected. The junior investigators who had received unified training conducted the survey to animal bite victims consulting the RPCs. The senior investigators checked the collected questionnaires daily to perform quality control. Data were double-entered into Epidata 3.0 separately by two individuals. A total of 1080 bite victims were interviewed, of which 65 individuals refused to answer all questions. The final analysis was undertaken on 1015 questionnaires.

### Measurement variable

The dependent variable was the exposure category. According to the severity of the wound, the WHO categorizes the animal bite as “category I” (touching/feeding of animals or licks on intact skin), “category II” (nibbling of uncovered skin, minor scratches or abrasions without bleeding), and “category III” (single or multiple transdermal bites or scratches, licks on broken skin, contamination of mucous membrane with saliva from licks) [6]. We investigated the exposure category of animal bite victims according to the guidelines from the WHO. Considering that animal bite victims defined as category I exposures need not initiate post-exposure prophylaxis (PEP), we excluded these victims and explored the influencing factors of category II and III exposures. The variable was coded as follows: 0 = “category II” and 1 = “category III.”

### Key predictors

#### Potential covariates

Demographic variables included age (1 = “1–15 years old,” 2 = “16–30 years old,” 3 = “31–45 years old,” 4 = “46–60 years old,” and 5 = “61 years old and above”), gender, and education (0 = “senior school and below” and 1 = “university and above”). The habit of playing with animals (0 = “yes” and 1 = “no”) was obtained through self-report.

#### Animal injury history

Participants were asked to indicate the type of the offending animal. Responses were categorized as 1 = “own domestic animal,” 2 = “domestic animal of other people,” and 3 = “stray animal.” The exposure-to-risk situations were classified as 1 = “improper care of animals,” 2 = “excessive play with animals,” 3 = “insufficient preparedness,” and 4 = “unprovoked aggression.” Definitions of the four situations were reported in a previous paper published by our research team [13]. In the analysis, each of the corresponding risk types was coded as follows: 0 = absence of the risk type and 1 = presence of the risk type.

#### Knowledge of rabies

Questions regarding knowledge on rabies included: the source of rabies virus transmission, the route of transmission, and rabies fatality. Responses were coded as: 0 = “wrong answer or do-not-know” and 1 = “right answer.”

### Statistics analysis

All statistical procedures were performed using the Statistical Analysis System (SAS) 9.4 for Windows (SAS Institute Inc., Cary, NC, USA). Descriptive analysis was carried out for all variables. Logistic regression analysis was used to identify the factors related to exposure categories (demographic variables, animal injury history, and knowledge of rabies). First, crude odds ratios (ORs) and 95% confidence intervals (95% CIs) for each independent variable were calculated using univariate logistic regression. Second, adjusted ORs and 95% CIs were calculated using multivariate logistic regression analysis. All comparisons were two-tailed, and *p*-values less than 0.05 were considered statistically significant.

## Results

Table 1 presents the characteristics of the 1015 animal bite victims attending the RPCs. Overall, the majority of bite wounds were category III exposures (55.57%), followed by category II exposures (41.18%) and category I exposures (3.25%). The mean age of these victims was 39.72 (standard deviation, 15.93), and 55.67% were female. More than half (56.85%) of respondents liked playing with animals, and 37.64% were hurt by animals at least twice. The most common sites of animal bites were the upper extremities (52.51%), followed by lower extremities (40.49%). Approximately one-third of injuries were attributed to unprovoked aggression (31.72%), followed by excessive play with animals (27.49%) and insufficient preparedness (26.70%), and the remaining 14.09% of the injuries were caused by improper care of animals. About 60% of the biting animals were stray animals or owned by other people, and 40% of animals involved in the injuries were owned by the victims. Of these biting animals, only 391 (38.52%) had previously received rabies vaccination.

**Table 1:** The characteristics of animal bite victims

Characteristic	Total	Exposure Category		
		Category I	Category II	Category III
	N (%)	N (%)	N (%)	N (%)
**Total**	1015	33 (3.25)	418 (41.18)	564 (55.57)
**Gender**				
Female	565 (55.67)	19 (57.58)	241 (57.66)	305 (54.08)
Male	450 (44.33)	14 (42.42)	177 (42.34)	259 (45.92)
**Age group**				
1–15 years old	168 (16.55)	6 (18.18)	96 (22.97)	66 (11.70)
16–30 years old	365 (35.96)	9 (27.27)	147 (35.17)	209 (37.06)
31–45 years old	176 (17.34)	4 (12.12)	72 (17.22)	100 (17.73)
46–60 years old	196 (19.31)	11 (33.33)	69 (16.51)	116 (20.57)
61 years old and above	110 (10.84)	3 (9.09)	34 (8.13)	73 (12.94)
**Educational level**				
Senior school and below	551 (54.28)	21 (63.64)	215 (51.44)	315 (55.85)
University and above	464 (45.71)	12 (36.36)	203 (48.56)	249 (44.15)
**Habit of playing with animals**				
Yes	577 (56.85)	26 (78.79)	259 (61.96)	292 (51.77)
No	438 (43.15)	7 (21.21)	159 (38.04)	272 (48.23)
**Frequency of animal bites**				
Once	633 (62.36)	20 (60.61)	254 (60.77)	359 (63.65)
Twice or more	382 (37.64)	13 (39.39)	164 (39.23)	205 (36.35)
**Exposure-to-risk situations**				
Improper care	143 (14.09)	4 (12.12)	40 (9.57)	99 (17.55)
Excessive play	279 (27.49)	7 (21.21)	132 (31.58)	140 (24.82)
Insufficient preparedness	271 (26.70)	8 (24.24)	123 (29.43)	140 (24.82)
Unprovoked aggression	322 (31.72)	14 (42.43)	123 (29.42)	185 (32.81)
**Offending animal**				
Own domestic animals	394 (38.82)	4 (12.12)	147 (35.16)	243 (43.09)
Domestic animals of other people	489 (48.18)	20 (60.61)	213 (50.96)	256 (45.39)
Stray animals	132 (13.00)	9 (27.27)	58 (13.88)	65 (11.52)
**Bite Location**				
Head and face	42 (4.14)	0 (0.00)	18 (4.31)	24 (4.25)
Lower extremities	411 (40.49)	21 (63.64)	188 (44.98)	202 (35.82)
Upper extremities	533 (52.51)	12 (36.36)	199 (47.61)	322 (57.09)
Torso	29 (2.86)	0 (0.00)	13 (3.11)	16 (2.84)
**Animal status**				
Vaccinated against rabies	391 (38.52)	22 (66.67)	180 (43.06)	189 (33.51)
Unvaccinated against rabies or unclear	624 (61.48)	11 (33.33)	238 (56.93)	375 (66.49)

Table 2 presents the factors associated with the severity of animal bites. The results of the univariate logistic regression model suggested that category III exposures were significantly associated with age, the habit of playing with animals, exposure-to-risk situations, the type of the offending animal, bite location, animal status, and knowledge of rabies transmission route. After controlling for confounders, the results of the multivariate logistic regression model showed that, compared with people aged less than15 years old, older respondents were more likely to be injured seriously (OR = 2.19, 95% CI: 1.45–3.30 for age group 16–30, OR = 2.06, 95% CI: 1.29–3.31 for age group 31–45, OR = 2.22, 95% CI: 1.39–3.55 for age group 46–60 and OR = 2.86, 95% CI: 1.65–4.97 for age group ≥61). People who had a habit of playing with animals (OR = 1.63, 95% CI: 1.23–2.17), those who were hurt by their own domestic animals (OR = 1.55, 95% CI: 1.14–2.10), victims exposed to animals unvaccinated against rabies (OR = 1.45, 95% CI: 1.08–1.95), and respondents who did not know that rabies is fatal once the clinical signs are manifested (OR = 1.40, 95% CI: 1.05–1.86) had higher odds for category III exposures. Compared to people bitten for unprovoked aggression, those exposed for insufficient preparedness had a greater risk for severe injures (OR = 1.53, 95% CI: 1.07–2.18). Respondents who had been bitten on at least two occasions (OR = 0.73, 95% CI: 0.55–0.97) were less likely to be injured seriously.

**Table 2:** Logistic regression analysis of factors associated with category III exposures (*N* = 982^*^)

Characteristic	Crude OR ^**b**^	95% CI ^**c**^	***P***	Adjusted OR	95% CI	***P***
**Gender (Ref.** ^**a**^ **= Female)**						
Male	1.16	0.90–1.49	0.2647	1.30	0.99–1.70	0.0570
**Age group (Ref. = 1–15 years old)**						
16–30 years old	2.07	1.42–3.02	0.0002	2.19	1.45–3.30	0.0002
31–45 years old	2.02	1.31–3.12	0.0016	2.06	1.29–3.31	0.0026
46–60 years old	2.51	1.63–3.86	<.0001	2.22	1.39–3.55	0.0008
61 years old and above	2.99	1.79–5.02	<.0001	2.86	1.65–4.97	0.0002
**Educational level (Ref. = University and above)**						
Senior school and below	1.24	0.96–1.60	0.0953	1.25	0.93–1.67	0.1336
**Habit of playing with animals (Ref. = No)**						
Yes	1.53	1.18–1.98	0.0013	1.63	1.23–2.17	0.0008
**Improper care (Ref. = No)**						
Yes	2.54	1.63–3.97	<.0001	1.64	0.98–2.75	0.0621
**Excessive play (Ref. = No)**						
Yes	1.39	1.05–1.83	0.0231	0.70	0.48–1.02	0.0615
**Insufficient preparedness (Ref. = No)**						
Yes	0.79	0.59–1.04	0.0965	1.53	1.07–2.18	0.0192
**Unprovoked aggression (Ref. = No)**						
Yes	1.13	0.86–1.48	0.3846	–	–	–
**Offending animal (Ref. = Domestic animals of other people or stray animals)**						
Own domestic animals	1.55	1.16–2.07	0.0028	1.55	1.14–2.10	0.0051
**Head and face (Ref. = No)**						
Yes	1.03	0.56–1.91	0.9239	–	–	–
**Lower extremities (Ref. = No)**						
Yes	0.72	0.55–0.93	0.0107	0.89	0.43–1.84	0.7609
**Upper extremities (Ref. = No)**						
Yes	1.48	1.15–1.90	0.0027	1.34	0.66–2.71	0.4227
**Torso (Ref. = No)**						
Yes	0.91	0.43–1.91	0.8015	1.39	0.84–1.85	0.4086
**Animal status (Ref. = Vaccinated against rabies)**						
Unvaccinated against rabies or unclear	1.50	1.16–1.95	0.0023	1.45	1.08–1.95	0.0139
**Frequency of animal bites (Ref. = Once)**						
Twice or more	0.88	0.68–1.15	0.3558	0.73	0.55–0.97	0.0295
**Knowledge of the source of transmission (Ref. = Yes)**						
No	0.98	0.76–1.26	0.8563	0.87	0.63–2.31	0.3149
**Knowledge of the route of transmission (Ref. = Yes)**						
No	1.84	1.09–3.11	0.0233	1.60	0.90–2.83	0.1073
**Knowledge of rabies fatality (Ref. = Yes)**						
No	1.28	0.99–1.66	0.0593	1.40	1.05–1.86	0.0207

^*^ Total number < 1015 because we exclude 33 (3.25%) bite victims with category I exposures

^a^ Ref, reference; ^b^ *OR* Odds ratio; ^c^ *CI* Confidence interval

## Discussion

Animal bites are an important public health problem. The present study focused on the wound severity of bite victims. Results showed that people who were hurt by their own domestic animals, victims without the knowledge of rabies fatality, and those exposed to animals unvaccinated against rabies had a higher risk for category III exposures. In addition, this study found that respondents aged 16–30 years old were the most common victims. The findings may help to improve the national strategy for preventing animal bites and reduce the related health and economic burden.

Early management of bite wounds generally guarantees the prevention of disease progression. According to the WHO recommendations, no medical care is needed for category I exposures. However, victims who were category I exposures in the present study also visited rabies prevention clinics for medical assistance, which indicated that some people were less aware of the prerequisites for PEP initiation. Therefore, it may be necessary to enhance health education about PEP among the public. Our study found that people without knowledge of rabies fatality had a higher risk for category III exposures compared to those who knew that rabies is fatal. This may suggest that improving public knowledge of rabies would be helpful to reduce severe animal bites.

The present study showed that people aged 16–30 years were bitten more than other age groups, while previous studies reported that children aged less than 15 years old were the most common victims [14–16]. This may be due to the relatively small number of children in the population. Additionally, owing to the implementation of the National Family Planning Policy (the one-child policy before 2016) in China, most families have only one child, especially in urban areas. Parents and other family members take more care of their child, which can protect children from animal attacks to a certain extent. It is noteworthy that older people had a higher risk of being injured seriously compared with younger people. The limited motor skills to provide defense may explain this. In general, older people are less capable of dealing with animal attacks than younger people. Recommendations for parents, children, and dog-owners on how to avoid animal attacks should be given.

The majority of animal bite events are attributable to domestic animals in our study. This is in line with previous reports from Sri Lanka [17] and Iran [18] but is inconsistent with other studies, which reported that stray animals accounted for most bites [16, 19, 20]. People who were hurt by their own domestic animals were more likely to be injured seriously compared with those bitten by domestic animals of other people or stray animals. Therefore, there is a need to increase awareness to prevent bites by domestic animals (mainly dogs and cats) in China. Since dogs and cats can serve as a link for rabies virus between wildlife and humans, vaccination of dogs and cats continues to be an effective public health measure in preventing rabies in humans [21]. However, the vaccination rate of the biting animals was less than half in our study. Similar findings were also reported in Kenya [20]. Victims exposed to animals vaccinated against rabies had a lower risk for severe injures. Therefore, campaigns for rabies vaccination of dogs and cats are urgently needed.

In the present study, the regions of the human body more vulnerable to animal bites were upper extremities, and this is consistent with a study conducted in Iran [18]. However, most previous studies reported that lower limbs were the major site of animal bites, especially the feet [7, 20, 22]. It may be attributed to the different exposure-to-risk situations. In general, the bites on the lower extremities are mainly owing to escaping from the aggressive animal, while the bites on the upper extremities are more caused by playing with them [7]. Furthermore, the study found that the severity of animal bites had no significant difference among different bite sites. Notably, the bites occurring to the head and face are especially risky due to proximity to the central nervous system, and clinical progression to rabies is highly likely if bitten by a rabid animal [20]. Such victims should receive immediate PEP to prevent any possibility of developing rabies.

There is a potential rabies exposure from any bite or scratch wound by a suspected rabid animal. Generally, as long as there are slight teeth marks or scratches on the skin, timely and appropriate prophylaxis measures should be taken. Studies have reported that minor scratches without bleeding can also cause rabies [23, 24]. Therefore, it is essential to raise awareness among the public about rabies risk, modes of transmission, and exposure categories of wounds, which may help to urge victims to seek medical attention.

Several limitations need to be acknowledged. First, there are different classification criteria about the severity of bite wounds, the present study investigated the exposure category of animal bite victims according to the guidelines from the WHO, and the findings may be more comparable and of certain reference value for other countries. Second, the study site is mainly in an urban area, therefore, the research results may not be extended to rural areas. Third, we failed to follow the status of rabies among these victims or biting animals. Further prospective studies are needed to track the health outcomes of victims with different exposure categories after initiating PEP and explore the relationship between wound exposure categories and rabies.

## Conclusions

In summary, the present study showed that the severity of bite wounds in central China was associated with age, the habit of playing with animals, exposure-to-risk situations, types of the offending animal, animal status, and knowledge of rabies fatality. In order to reduce the serious animal bite and the risk of developing rabies, health education about how to respond to animal attacks and rabies PEP should be conducted, and, more importantly, the necessity and importance of vaccinating owned dogs and controlling free-roaming owned dogs and stray dogs for disease prevention and elimination should be emphasized. In addition, most animal bites are attributable to domestic animals. The need to improve community awareness of preventing bites by domestic animals should be highlighted.

## Data Availability

The datasets used in the current study are available from the corresponding author on reasonable request.

## Abbreviations

WHO: World Health Organization
RPCs: Rabies prevention clinics
PEP: Post-exposure prophylaxis
OR: Odds ratio
CI: Confidence interval
